# Neural Bases of Unconscious Error Detection in a Chinese Anagram Solution Task: Evidence from ERP Study

**DOI:** 10.1371/journal.pone.0154379

**Published:** 2016-05-05

**Authors:** Hua-zhan Yin, Dan Li, Junyi- Yang, Wei Li, Jiang Qiu, Ying-yu Chen

**Affiliations:** 1 Department of Psychology, Chongqing Normal University, Chongqing, 401331, China; 2 School of Psychology, Southwest University (SWU), Chongqing, 400715, China; Liaoning Normal University, CHINA

## Abstract

In everyday life, error monitoring and processing are important for improving ongoing performance in response to a changing environment. However, detecting an error is not always a conscious process. The temporal activation patterns of brain areas related to cognitive control in the absence of conscious awareness of an error remain unknown. In the present study, event-related potentials (ERPs) in the brain were used to explore the neural bases of unconscious error detection when subjects solved a Chinese anagram task. Our ERP data showed that the unconscious error detection (UED) response elicited a more negative ERP component (N2) than did no error (NE) and detect error (DE) responses in the 300–400-ms time window, and the DE elicited a greater late positive component (LPC) than did the UED and NE in the 900–1200-ms time window after the onset of the anagram stimuli. Taken together with the results of dipole source analysis, the N2 (anterior cingulate cortex) might reflect unconscious/automatic conflict monitoring, and the LPC (superior/medial frontal gyrus) might reflect conscious error recognition.

## Introduction

The brain needs to continuously monitor behavior in real time to accomplish specific tasks. When the results of behavior are not on target, namely as erroneous behavior occurs, the brain detects the error and makes adjustments to subsequent behavior in order to reach the expected goal and thus fit the environment better[1–4]. The error monitoring system helps to correct immediate behavioral faults[5].

Many researchers have been suggesting that error processing might be executed at different levels of consciousness, and the processing of error detection and error awareness occurs at different levels in the error monitoring system. Specifically, error detection occurs when an error is detected by the brain but the subject is unaware of the error, whereas error awareness is defined as the ability to explicitly report the error verbally or press a key to indicate the error [4,6–8]. Typically, we make a mistake but are not aware of it, so researchers pay more attention to error detection.

Event-related potentials (ERPs) have made it possible to precisely record the spatiotemporal cortical activation patterns associated with error detection. Most commonly, the ERP methods employed for this purpose analyze response-locked and stimulus-locked electrophysiological activity. In response-locked electrophysiological activity, for example, previous ERP studies have indicated that error negativity (Ne/ERN) and error positivity (Pe) might be associated with error monitoring and error detection [4,7,9–12]. Specifically, the ERN is a negative deflection with a fronto-central maximum that peaks 50–100 ms after an erroneous response in choice reaction time tasks [13–16]. The Pe is a positive deflection that has a centro-parietal maximum peaking at 200–400 ms, and frequently follows the ERN after an erroneous response [17,4]. Some studies have also suggested that the ERN is elicited irrespective of whether or not the subject is aware of the error [18–21], whereas the Pe is associated with conscious error recognition [20,22]. In stimulus-locked electrophysiological activity, Britz and Michel investigated the temporal dynamics of the scalp topography of stimulus-locked high-density ERPs elicited by errors and correct trials in the Stroop task, and showed amplitude differences between error and correct trials in two time windows around 200 ms and 400 ms after stimulus onset, but these differences in source strength did not reach statistical significance[23].

Previous ERP studies have mainly focused on response-locked electrophysiological activity in error processing, and have found that ERN and Pe were related to error processing. However, in our study, we have tried to explore stimulus-locked electrophysiological activity instead of response-locked brain activity.

With the development of brain imaging technology, research has increasingly focused on the activation patterns of brain areas associated with error processing. For example, some studies have provided direct evidence that the mPFC/ACC are activated when subjects make an erroneous response without being consciously aware of it [3–4,24]. Others studies have found that the anterior cingulate cortex (ACC) might be involved in the conflict monitoring mechanism during information processing [25–28]. A number of studies have found that activation of the medial frontal gyrus is related to conscious cognitive control [29–30].

Therefore, in the present study, we used high-density (64-channel) ERPs combined with dipole source analysis to explore the neural bases of unconscious error detection (i.e., detecting an error without a conscious awareness of the error being detected) in an anagram solution task. As in an English anagram task (e.g., the solution for “oxmia” is “axiom” by rearranging the letters) [31], we asked our subjects to reconstruct a set of wrongly arranged radicals into a Chinese character (a radical is a basic component of a Chinese character, and a character is typically made of several radicals arranged in a prescribed pattern; for more detail, see the Materials section). We were interested in the unconscious error detection (UED) response, the no error (NE) response, and the detect error (DE) response. In the NE response, the anagram was a lexical anagram (hence could be reconstructed into a correct Chinese character), and subjects correctly responded “yes” to the anagram and did not change the response (meaning that they determined that the anagram could be reconstructed into a legitimate Chinese character). The UED response was defined as an explicit “yes” response to a nonlexical anagram (which contained an incorrect radical and hence could not be converted into a correct Chinese character) with no subsequent change in the response. The DE response was defined as a “no” response to a nonlexical anagram (which contained an incorrect radical and hence could not be converted into a correct Chinese character) and no change in the response. Thus, for the UED response type, on an explicit level, subjects actually missed the error radical in the nonlexical anagram, but at the neurological or implicit level, the response differed from the NE and DE response, suggesting that they detected the error radical or identified the nonlexical anagram unconsciously. Consequently, we hypothesized that the average N2 amplitude [32–35]and the intensity of ACC activation) [3–4,24] in the UED condition are higher than those in the NE and DE conditions, and the average LPC amplitude and activation intensity of the superior/medial frontal gyrus in the DE condition are higher than those in the UED and NE conditions [36–38].

## Method

### Participants

Twenty undergraduates (11 women, 9 men) aged 19 to 24 (mean age 20.8 years, *SD* = 1.58) at Southwest University in China participated as paid volunteers. They were right-handed, had normal or corrected-to-normal vision, and no current or past neurological or psychiatric disorders. This study was approved by the IRB at Southwest University. Subjects all gave their written informed consent before the experiment started.

### Materials

**Fig 1:**
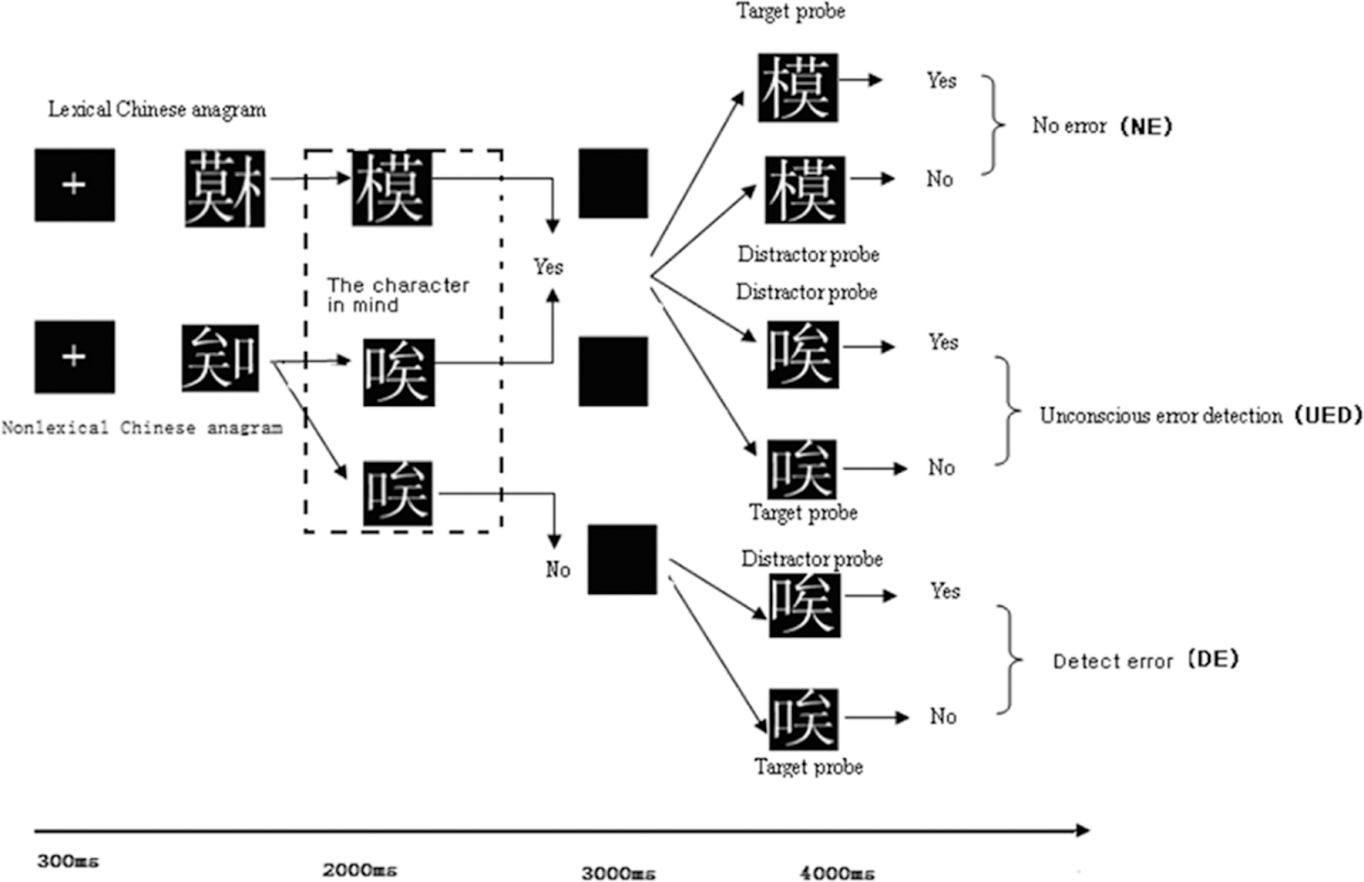
Flow of stimuli in each trial of the ERP experiment. There were two different trial types: lexical Chinese anagrams and nonlexical Chinese anagrams. In the formal experiment, a lexical Chinese anagram or nonlexical Chinese anagram was presented to the subjects; and subjects then decided whether the anagram could be reconstructed into a correct Chinese character. A correct or incorrect Chinese character was subsequently presented as a probe to determine the anagram solution of the subject. Subjects were required to judge whether the character they generated was the same as the probe character. No error (NE) condition means that there was no error in the stimulus, and the subjects responded correctly, so no error was detected. Unconscious error detection (UED) means that subjects were not aware of having generated an incorrect character when a nonlexical Chinese anagram appeared. By contrast, the detect error (DE) condition meant that subjects detected that the nonlexical Chinese anagram could not be reconstructed into a correct Chinese character.

In a logographic language such as Chinese, characters are composed of radicals, which in turn, are composed of strokes. Chinese characters are normally written using a fixed stroke sequence (e.g., which stroke is drawn first, and which second stroke is prescribed). Radicals usually consist of several strokes and are arranged or combined in a prescribed way to form a Chinese character. Generally, Chinese characters can be broken down into components or radicals. For example, the Chinese character “xiang (think)” (想) is made up of three radicals, “mu” (木), “mu” (目) and “xin” (心). In the formal experiment, Chinese anagrams were presented for subjects to solve. The anagram was created from a character by scrambling or rearranging the radicals that make up the character. Two types of anagrams were used, *lexical* and *nonlexical* anagrams. The radicals of a lexical anagram were intact radicals from a character that can be used as building blocks to reconstruct the original character. A nonlexical anagram contained one radical that was altered from the radical in the original character, for example, by adding one stroke to, or removing one stroke from the original radical. This makes it impossible to reconstruct the anagram into a lexical character since one “building block” is altered. In our study, there were no significant differences in operating frequency and strokes of the Chinese characters described as nonlexical and lexical. The changes were very minor and not easily noticeable (For more details, see Fig 1). Subjects were presented with either a lexical or a nonlexical anagram and required to determine as fast as they could whether they could reconstruct the anagram into a lexical character. It was the Chinese counterpart of solving, for example, the English anagram of “oxmia” by rearranging the letter into “axiom” [31].

### Design and Procedure

In the experiment, subjects were seated in a semi-dark room facing a computer screen positioned 70 cm from their eyes. The experiment was divided into a practice phase and a test phase. When the subject was familiar with the procedure of the experiment, the test phase started. The formal ERP test consisted of six blocks, and every block had 44 judgment trials (22 trials of lexical anagrams, 22 trials for nonlexical anagrams) presented in random order. The whole experiment had 264 trials (132 lexical anagrams, 132 nonlexical anagrams). Subjects were instructed to keep their eyes fixated on the screen, and avoid blinking or looking down at their fingers during the task performance, or making movements of any sort. They could take a short break after finishing each block. In each trial, the characters were presented at the center of the screen in Song Ti font with a size of 72 points. The sequence of events is shown in Fig 1. After a 300-ms fixation point display, a Chinese anagram (visual angle, 2.86°) was presented until the subject pressed the response key or 2000 ms expired (whichever came first). They were instructed to press the “1” key (“Yes” response) if they thought that the Chinese anagram could be reconstructed into a correct (i.e., lexical) character, or press the “2” key (“No” response) if they thought that the Chinese anagram could not be reconstructed into a correct character, or to make no response if they could not decide whether the Chinese anagram could be reconstructed into a correct character. In half of the trials, the anagram was a nonlexical anagram. In the other half of the trials, the anagram was a lexical anagram. After the response was made, a blank screen appeared for 3000 ms, unless the subject reversed the selection and pressed the response key. After the blank screen, a target probe or a distracter probe (visual angle of 2.86°) would remain until the subject pressed a key within 4000 ms. A target probe was a reconstructed “character” version of the earlier displayed anagram keeping the original radicals in the anagram intact regardless of whether the anagram was lexical or nonlexical (in which one of the radicals was altered). A distractor probe was one in which one radical in the anagram was changed from its original form, either from a correct to an incorrect or from an incorrect to a correct one. If the subjects responded “yes” to the anagram and did not change their response, they needed to respond to the probe to indicate whether their reconstructed character was the same as the probe by pressing “1” for “yes” or “2” for “no”. If they responded “no” or did not respond to the anagram, they needed to indicate whether the probe was a lexical or nonlexical character by pressing “1” for “yes” or “2” key for “no” (for more details, see Fig 1). In half of the trials, the probes were target probes. In the other half of the trials, the probes were distractor probes. To illustrate the test procedure, suppose a lexical anagram was displayed and a subject correctly judged that it could be reconstructed into a lexical character and did not correct the response. In that case, when the probe was displayed, the subject should have responded “yes” to a target probe, and “no” to a distractor probe. Now, suppose a nonlexical anagram was displayed and the subject failed to detect the altered radical in the anagram and did not correct the response. In that case, when the probe was displayed, he or she should respond “yes” to a distractor probe (showing the corrected radical instead of the incorrect radical in the anagram) and “no” to a target probe (showing the original incorrect radical). Half of the subjects were instructed to press “1” for “yes” and press “2” for “no”. The other half of subjects were instructed to press “2” for “yes” and press “1” for “no”.

In our study, we were interested in no error (NE), unconscious error detection (UED), and detect error (DE) response types. The UED response was an unconsciousness error response to a nonlexical anagram stimulus, but the DE and NE were correct responses to nonlexical and lexical anagram stimuli. Our aim was to study the neural bases of unconsciousness error detection, so we could distinguish the difference between unconsciousness error responses and correct responses elicited by different stimuli or cognitive processing when we compared UED with DE and NE responses. Specifically, the NE response meant that subjects responded “yes” to a lexical anagram and did not change their response, and subsequently responded “yes” to a target probe (a correct Chinese character) or “no” to a distractor probe (an incorrect character). Thus, in this response type, there was no error in the stimulus, and the subjects’ response was consistent with the stimulus. By contrast, the UED response meant that subjects responded “yes” to a nonlexical Chinese anagram, were apparently unaware of the nonlexical feature (the altered radical) in the anagram, and did not change their response. In other words, they believed that they had generated a correct character from the radicals of the nonlexical Chinese anagram and did not correct their response. Thus, this response would be classified as a UED if subjects responded “yes” to the nonlexical anagram and did not correct the response, and subsequently responded “yes” to the distractor probe (a correct character), or responded “no” to the target probe (an incorrect Chinese character). The DE response type meant that subjects responded “no” to a nonlexical anagram and did not change their responses, and subsequently responded “no” to a target probe (an incorrect Chinese character) or “yes” to a distractor probe (a correct character).

### Electrophysiological recording and analysis

Brain electrical activity was recorded from 64 scalp sites using tin electrodes mounted in an elastic cap (Brain Products, Gilching, Germany), with the average reference electrode on the left and right mastoids and a ground electrode on the medial frontal aspect. The vertical electrooculograms (EOGs) were recorded supra- and infra-orbitally at the left eye. The horizontal EOG was recorded from the left versus right orbital rim. All interelectrode impedance was maintained below 5 kΩ. The EEGs and EOGs were amplified using a 0.05–80 Hz bandpass and continuously sampled at 500 Hz/channel for offline analysis. Artifacts from eye-blink movements were rejected offline. Trials with EOG artifacts (where the mean EOG voltage exceeded ± 100 μV) and those contaminated with artifacts due to amplifier clipping, bursts of electromyographic activity, or peak-to-peak deflections exceeding ± 100 μV were excluded from averaging.

We analyzed the ERPs elicited by UED, NE, and DE responses. Our principal analysis was of the ERP elicited by the Chinese anagrams. The averaged epoch for an ERP was 1600 ms, including 1400 ms post-stimulus and 200 ms pre-stimulus. As observed in the grand averaged waveforms, the ERPs elicited by the UED, DE, and NE responses were clearly distinct from each other. All these differences were prominent over the front-central and central-parietal regions of the scalp. Thus, the following six electrode points were chosen for statistical analysis within the 300–400-ms time window: F1, Fz, F2, FC1, FCz, and FC2. The following four electrode points were chosen for statistical analysis within the 900–1200-ms time window: P1, P2, CP1, and CP2. Mean amplitudes were analyzed using two-way repeated-measures Analysis of variance (ANOVA). The factors were response type (UED, DE, and NE) and electrode site. For all analyses, the *p* value was corrected for deviations based on the Greenhouse–Geisser procedure.

### Dipole source analysis

The Brain Electrical Source Analysis program (BESA Version 5.0 Software; BESA, Gräfelfing, Germany) was used to perform dipole source analysis, using the four-shell ellipsoidal head model. In order to focus on scalp electrical activity related to the processing of unconscious error detection, the averaged ERPs evoked by the DE were subtracted from the ERPs evoked by the UED in the 300–400-ms and 900–1200-ms time ranges. When the dipole points were determined, the software automatically determined the location of the dipoles. The relevant residual variance criterion was used.

## Results

### Behavioral data

The mean number of trials for NE, UED, and DE responses were 81 ± 5, 38 ± 6, and 40 ± 6, respectively. We recorded the reaction time (RT) from when the anagram appeared until a response was made. Mean RTs for the NE, UED, and DE were 1410 ± 66 ms, 1433 ± 75 ms, and 1498 ± 67 ms, respectively. A repeated-measures ANOVA of mean RTs showed that the main effect of response type was significant, *F*(2, 38) = 13.764, *p* < 0.001. Post-hoc testing showed that the mean RT of the UED response was significantly shorter than that of the DE response (*p* < 0.01) and the mean RT of the NE response was also significantly shorter than that of the DE response (*p* < 0.001). The nonlexical anagram that subjects thought could not be reconstructed into a correct character imposed an increasing load on the working memory in the DE response, so the RTs of the DE response were significantly longer than those of the UED and NE. However, there was no significant difference between the mean RTs of UED and NE responses (*p* = 0.092).

### Electrophysiological scalp data

**Fig 2:**
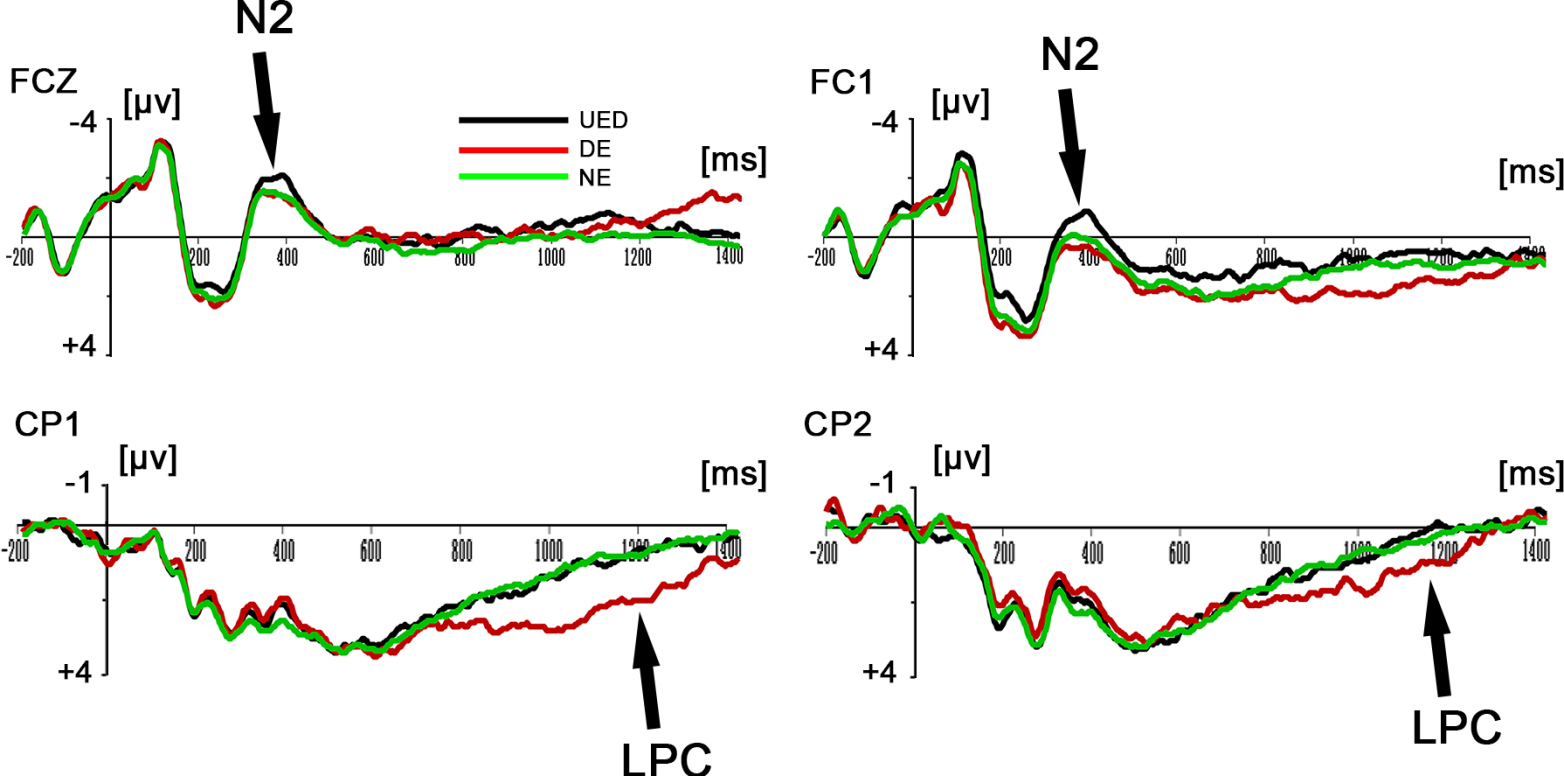
Grand-mean ERP waveforms of the N2 and LPC components. **Top:** Grand average ERPs FCZ, FC1 for the UED, DE and NE response types in the time window of 300-400ms. **Bottom:** Grand average ERPs CP1, CP2 for the UED, DE and NE response types in the time window of 900-1200ms.

**Fig 3:**
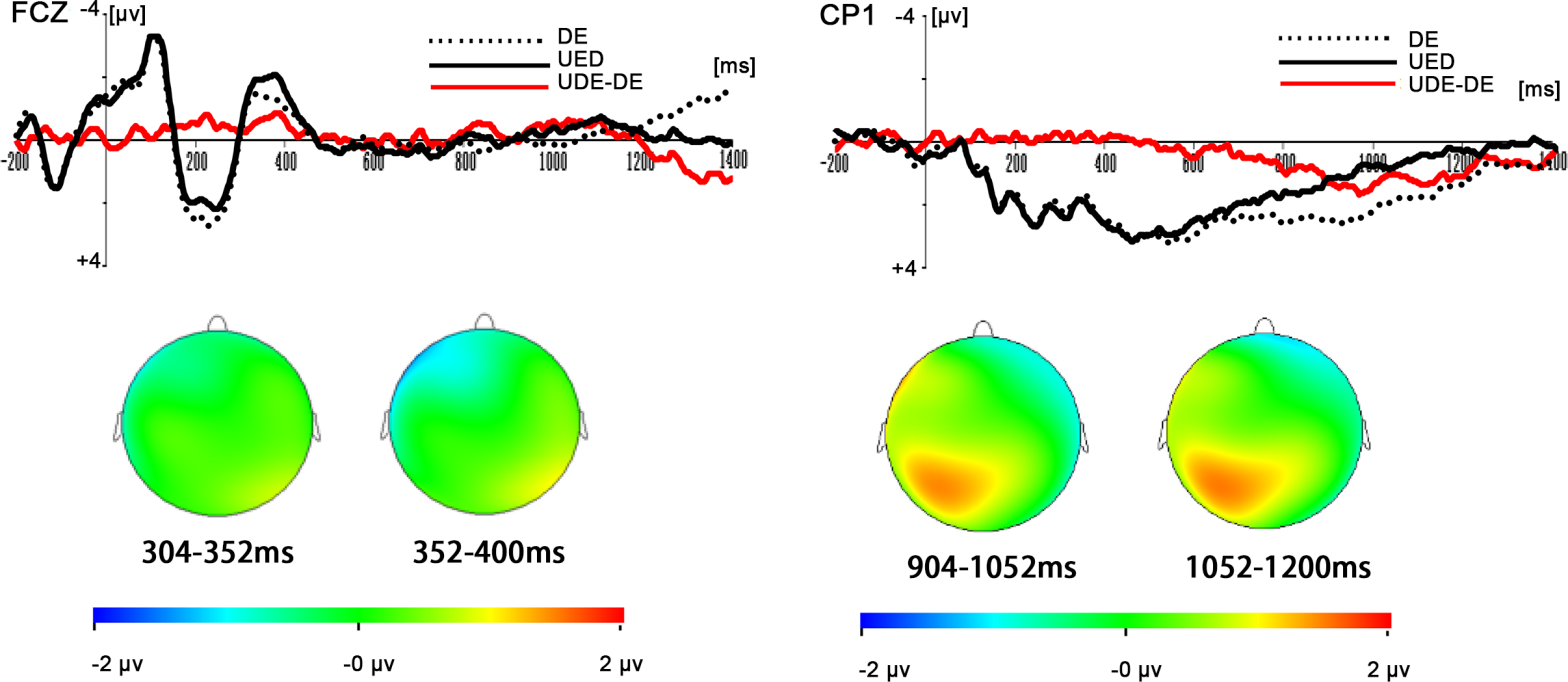
Grand-mean ERP difference waveforms and topographies of the N2 and LPC components. **Top:** Grand average ERPs FCz for the UED and DE conditions and difference waves (UED minus DE) in the time window of 300-400ms and CP1 for the UED and DE conditions and difference waves (DE minus UED) in the time window of 900-1200ms. **Bottom:** Topographical maps of the voltage amplitudes for the difference waves (UED minus DE) from 300 ms to 400ms and the voltage amplitudes for the difference waves (DE minus UED) from 900 ms to 1200 ms.

Results for two-way repeated-measures ANOVAs revealed that there was a significant main effect of response type for the main amplitude in the 300–400-ms time window, *F*(2, 38) = 4.121, *p* < 0.05. Post-hoc tests showed that the UED elicited a more negative ERP component than did the DE (*p <* 0.05) and the NE (*p <* 0.05) (see Fig 2). In addition, the results of the ANOVAs showed that the main effect of response type for the main amplitude in the 900–1200-ms time window was also significant, *F*(2, 38) = 9.22, *p* < 0.01. Post-hoc tests showed the DE elicited a significantly greater late positive component (LPC) than did the UED (*p <* 0.05) and the NE (*p <* 0.001; see Fig 2 and Fig 3).

**Fig 4:**
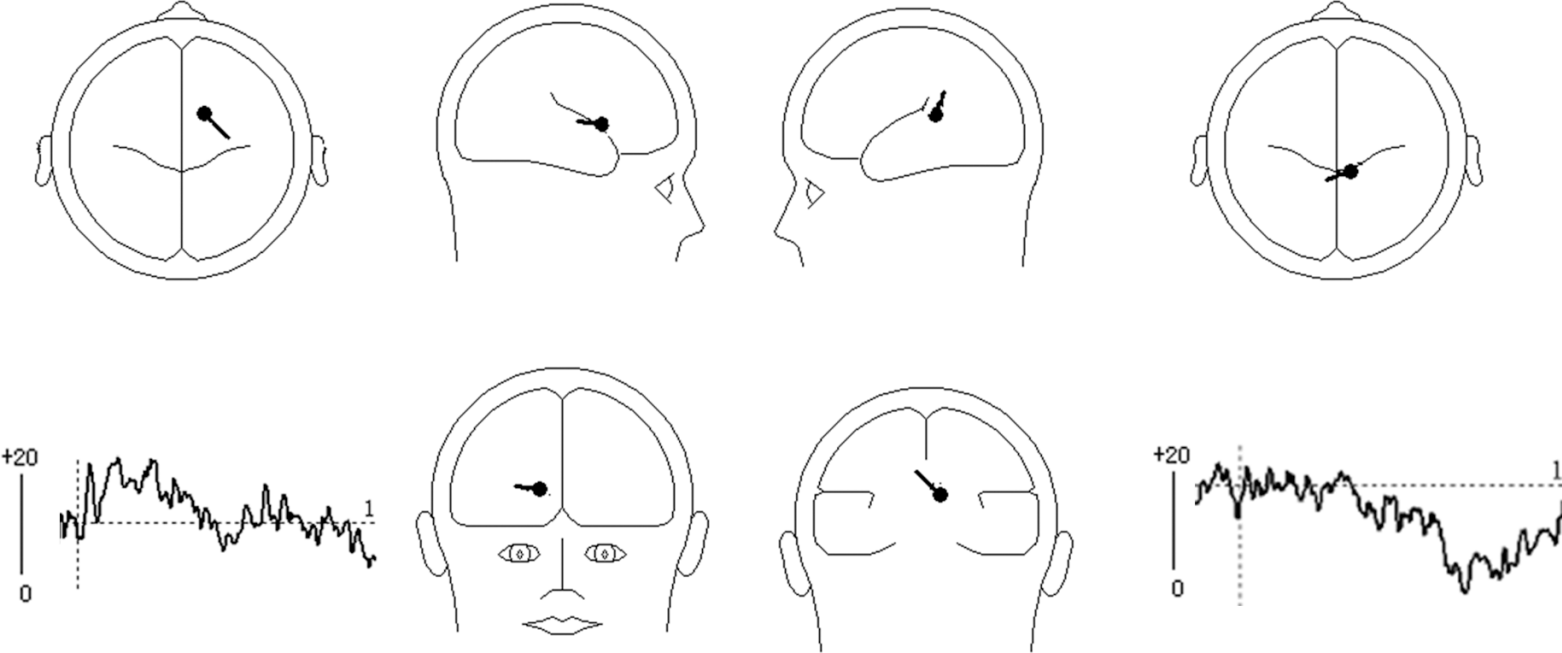
Results of dipole source analysis of the difference waves (UED minus DE) in the N2 and LPC components. The left-bottom and right-bottom images show the source activity waveforms; the figures display the mean locations of the dipoles. **Left:** In the time range of 300–400 ms, the dipole was located near the cingulate gyrus (BA32: x = 14.4, y = 23.5, z = 42.8); **Right**: In the time range of 900–1200 ms, the dipole was located near the middle frontal gyrus (BA6: x = 9.2, y = -15.1, z = 51.3)

BESA Software was used to perform dipole source analysis. In order to explore and increase the precision of source locations, principal component analysis (PCA) was conducted on the ERP difference waves for UED minus DE (64 channels). Firstly, PCA indicated that one component was needed to explain 100% of the variance in the data between 300–400 ms. Therefore, a dipole was fitted with no restriction on the direction and location. The result indicated that the dipole was located in the ACC (BA32: x = 14.4, y = 23.5, z = 42.8). This model best explained the data and accounted for most of the variance, with a residual variance (RV) of 18.7% (see Fig 4). Secondly, PCA indicated that one component was needed to explain 100% of the variance in the data between 900–1200 ms. Therefore, the dipole was fitted with no restriction on the direction and location. The result indicated that the first dipole was located near the middle frontal gyrus (GFm)/gyrus frontalis medialis (GFd) (BA6: x = 9.2, y = -15.1, z = 51.3). This model explained the data best and accounted for most of the variance, with a RV of 19.4% (see Fig 4).

## Discussion

In the present study, we were interested in brain electrical activity related to error detection that occurred during the solution of an anagram before erroneous responses were executed. That is to say, our ERP results focused on the stimulus-locked electrical activity. We showed that there were two ERP components (N300-400 and LPC) that were different in UED, DE, and NE responses.

At present, there are two possible interpretations of N300-400: one is semantic integration related to the N400, the other is the negative ERP component N2. First, the UED elicited a greater negative component (N2) in the 300–400-ms time window than did the NE and DE after the onset of the anagram. The N400 is a negative ERP component that peaks around 400 ms after word onset [32,39], so we could define the negative component in the 300–400-ms time window in our study as the N400. The N400 appears to be an index of semantic integration processes (Sitnikova et al., 2003)[40]. For example, the N400 is more negative in response to semantically incongruous words than congruous ones [39,41]. The N400 also has been described as reflecting how easily the word can be integrated into the current context) [39,42–43]. Perhaps the most widely accepted account of the N400 is that its amplitude is associated with the difficulty or mental effort required to integrate an item into the semantic context [40].

The N300–400 was actually the second negative component in the waveforms. Therefore, it might be an N2 component. The N2 is a negative ERP component peaking between 200 and 400 ms after stimulus onset and maximal at the fronto-central scalp location [32,44]. The N2 reflects the activity of the conflict-monitoring mechanism [44–46]. The N2 is typically larger after incongruent compared with congruent trials [47]and is larger in trials that include conflict between competing response representations, such as incongruent trials in the Stroop task [44,48]. However, some research has indicated that the N2 is increased in trials involving competing response activations but not in trials involving conflict in stimulus categorization [46,49], The N2 has also been proven to reflect conflict monitoring for correct responses[50]. Moreover, there is evidence that the N2 is also sensitive to the frequency of particular trial types [44], with the N2 amplitude being greater for low frequency trials [44,51–52]. The conflict monitoring function is essential for regulating many forms of everyday behavior [44] and the ACC is the crucial brain area that responds to conflict in information processing [53–54]. The ACC is thought to be associated with the selection and coupling of perceptual information to motor actions [34]. The amplitude of the N2 component related to response conflict is thought to be detectable by the ACC (Bartholow et al., 2009; Liotti et al., 2000; Van Veen & Carter, 2002)[55–56,48]. Based on these findings, we can infer from our study that the enhanced fronto-central N2 (conflict monitoring) component in the 300–400-ms time window located near the ACC indicated that conflict occurred in the UED response. Moreover, the UED response was an unconsciously incorrect response that elicited a more negative ERP component (N2) than did the correct NE and DE responses, so we can infer that the enhanced N2 in our study is also a signal of unconscious error detection.

Second, we also found that the DE elicited a greater LPC magnitude than did the NE and UED between 900–1200 ms. Previous studies have indicated that slow waves in the ERP are correlated with rehearsal/retention operations in the working memory [37–38]. Moreover, Berti et al. found that the larger the processing demands needed to keep object information in the working memory [57], the higher the slow wave activity (King & Kutas, 1995; Mecklinger & Pfeifer, 1996; Ruchkin et al., 1992)[37–38,58]. Similarly, Helenius et al. (2010) recorded stimulus-locked electrophysiological activity in a Go/NoGo task, and found that the NoGo correct trials elicited a prominent LPC after stimulus onset, as well as a positive deflection after the NoGo error trial peak, which was approximately 50 ms later than the LPC for the NoGo correct trials [59]. Increased perceptual effort was required in parietal association areas to encode the word characteristics of degraded words in nonverbal processes[36]. In our study, dipole analysis localized the generator of the LPC to the medial/superior frontal gyrus. A medial superior frontal gyrus (mSFG) region centered on the pre-supplementary motor area (pre-SMA) is thought to contribute to the higher cognitive function involved in task control, selection of actions [60], attention deployment[61], and recognition memory[62].

However, because the task for subjects was to judge whether a Chinese anagram could form a correct character, they tended to expect that the Chinese anagram would form a correct character. Subjects were required to analyze lexical Chinese anagrams and nonlexical Chinese anagrams in the NE and UED conditions, respectively, and then judge whether the characters were correct. In the DE condition, subjects were required to analyze a nonlexical Chinese anagram, and then judge whether or not it was a correct character, so they would once again evaluate and confirm their choice based on this tendency. Therefore, the increased LPC in the DE response type in our study might be related to re-evaluation and confirmation processes in the working memory when a stimulus did not form a correct character.

## Conclusions

In the present study, ERPs were used to examine the neural bases of stimulus-locked unconscious error detection. Our ERP data showed that the amplitude of the N2 elicited by the UED response was greater than those of the NE and DE responses, and the mean amplitude of the LPC elicited by the DE response was greater than those of the UED and NE responses. Together with the results of dipole source analysis, the N2 (in the anterior cingulate cortex) might reflect unconscious error detection in information processing. The LPC in the DE response in our study might be related to conscious error recognition.

## Supporting Information

S1 File The Average mapping of ERP for each subject in this study. (RAR)

S2 File The Grand Average mapping of ERPs for all subjects in this study. (RAR)
